# Efficacy and Safety of COVID-19 Treatment Using Convalescent Plasma Transfusion: Updated Systematic Review and Meta-Analysis of Randomized Controlled Trials

**DOI:** 10.3390/ijerph191710622

**Published:** 2022-08-25

**Authors:** Hyun-Jun Lee, Jun-Hyeong Lee, Yejin Cho, Le Thi Nhu Ngoc, Young-Chul Lee

**Affiliations:** 1 Department of BioNano Technology, Gachon University, 1342 Seongnam-Daero, Sujeong-Gu, Seongnam-si 13120, Gyeonggi-Do, Korea; 2 Department of Industrial and Environmental Engineering, Graduate School of Environment, Gachon University, 1342 Seongnam-Daero, Sujeong-Gu, Seongnam-si 13120, Gyeonggi-Do, Korea

**Keywords:** COVID-19, convalescent plasma transfusion, clinical outcomes, adverse events, systematic review, meta-analysis

## Abstract

This study investigated the efficacy and safety of convalescent plasma (CP) transfusion against the coronavirus disease 2019 (COVID-19) via a systematic review and meta-analysis of randomized controlled trials (RCTs). A total of 5467 articles obtained from electronic databases were assessed; however, only 34 RCTs were eligible after manually screening and eliminating unnecessary studies. The beneficial effect was addressed by assessing the risk ratio (RR) and standardized mean differences (SMDs) of the meta-analysis. It was demonstrated that CP therapy is not effective in improving clinical outcomes, including reducing mortality with an RR of 0.88 [0.76; 1.03] (I^2^ = 68% and *p* = 0.10) and length of hospitalization with SMD of −0.47 [−0.95; 0.00] (I^2^ = 99% and *p* = 0.05). Subgroup analysis provided strong evidence that CP transfusion does not significantly reduce all-cause mortality compared to standard of care (SOC) with an RR of 1.01 [0.99; 1.03] (I^2^ = 70% and *p* = 0.33). In addition, CP was found to be safe for and well-tolerated by COVID-19 patients as was the SOC in healthcare settings. Overall, the results suggest that CP should not be applied outside of randomized trials because of less benefit in improving clinical outcomes for COVID-19 treatment.

## 1. Introduction

The coronavirus disease 2019 (COVID-19) epidemic had spread from Wuhan, Hubei province, China, and subsequently rapidly became a pandemic since December 2019 [1]. According to a report by the World Health Organization dated 14 July 2022, 555,446,890 COVID-19 cases and 6,353,692 COVID-19-related deaths have occurred. The disease is defined as SARS-CoV-2 in that its angiotensin-converting enzyme 2 is bound to the surface of the lung cells, resulting in lung infection and subsequently acute respiratory symptoms or mortality [2]. In particular, SARS-CoV-2 contains four structural proteins, i.e., spikes, envelop, membranes, and nucleocapsids [2,3]. First, nucleocapsid proteins bind to the RNA and spike proteins mediate the virus attachment to the receptor of the host cell surface [3]. Next, the virus enters the cytoplasm of the host cell through the cleavage of the spike protein through a protease enzyme, after which the virus fuses with the cell membrane [2,3]. Subsequently, mature viruses are formed by synthesizing and translating the RNA, and then they are released to the cell surface via exocytosis, causing infection [3].

Recently, the COVID-19 disease has been well-controlled by governments and health organizations around the world based on widespread COVID-19 vaccination; however, specific drugs for COVID-19 have not been approved by Food and Drug Administration (FDA). However, in the early days of the pandemic, specific treatments and also vaccinations to prevent COVID-19 did not exist hitherto; therefore, the application of a number of antiviral agents (e.g., hydroxychloroquine/chloroquine (HCQ/CQ), arbidol, lopinavir-ritonavir, and remdesivir) and therapies (e.g., intravenous immunoglobulin, Chinese medicine, and convalescent plasma (CP) transfusion), which have been used to treat SARS and MERS, has been recommended [4]. Among these treatments, CP therapy, which transfuses plasma provided by previously infected individuals to infected patients, has been considered one of the most effective and safe treatments for treating COVID-19 patients compared with other conventional approaches [5,6]. In particular, if patients are infused at the appropriate time, then the patient can be prevented from becoming severely infected, thereby reducing their possibility of hospitalization in the intensive care units [7,8]. Recently, randomized controlled trials (RCTs) on the application of CP transfusion for COVID-19 treatment have been conducted in many regions of the world, and in fact, the effectiveness of this therapy has been extensively evaluated by several systematic reviews and meta-analyses of these RCTs. However, published meta-analysis articles that include only a small amount of eligible data may lead to an underestimation of the results. In addition, the number of RCTs on this topic has rapidly increased, so this updated systematic review and meta-analysis was designed to access a larger and higher quality data source in an attempt to provide a highly accurate result about this concern. In this study, clinical data were systematically collected and analyzed using meta-analysis to provide an overview and a statistical evaluation of the potential of CP transfusion against COVID-19. In particular, this meta-analysis not only assessed the effectiveness of CP transfusion in improving clinical symptoms and reducing all-cause mortality and length of hospitalization but also evaluated the risk of CP infusion-related adverse events compared with standard of care (SOC) or placebo therapy.

## 2. Materials and Methods

### 2.1. Literature Search Strategy

According to the Preferred Reporting Items for Systematic Reviews and Meta-analysis (PRISMA) protocol 2009 [9], a literature search pertaining to the efficacy and safety of COVID-19 treatment using CP transfusion was performed in the English language databases, i.e., Cochrane Library, PubMed, medRxiv, WHO International Clinical Trial Registry Platform, EMBASE, and Web of Science, to obtain relevant articles published up to 2022. The following keywords were used for the search: COVID-19, Coronavirus, SARS-CoV-2, CP, and RCT.

### 2.2. Study Selection and Data Extraction

We included all clinical trials that randomly assigned COVID-19 patients to CP plus SOC (intervention arm) therapy versus SOC or placebo (control arm). We considered all trials that randomized at least one patient in the intervention arm and one patient in the control arm, regardless of the treatment regime for CP or SOC or placebo, as long as there were no differences in the treatments used in the arms beyond the CP treatment or SOC or placebo. Trials of multiple arms were eligible if they directly compared CP versus SOC or placebo, with appropriate arms being included in the meta-analysis. Trials reported all-cause mortality, time to discharge from hospital, and adverse events on day 28 of the treatment regardless of whether it was the primary outcome. We did not make any restrictions on trial status, healthcare setting, and geographical region. On the other hand, non-RCTs, conference abstracts, presentations, reviews, basic science manuscripts, animal studies, and non-English articles were excluded from this systematic review. Moreover, repeated and similar studies were also not considered in this study.

Three independent reviewers screened each record of the included studies and extracted appropriate data for this meta-analysis. Extracted data included the title, author, country of study, number of participants, median age, intervention details (class and type of treatment), measurement outcomes (e.g., all-cause mortality, time to hospital discharge, and adverse events), and main results. The included data could be used to analyze the risk ratio (RR) or standardized mean differences (SMDs) of individual studies and finally provide meta-analysis results for the entire analysis.

### 2.3. Meta-Analysis

For all dichotomous outcomes (mortality and adverse events), the number of events and the number of total participants in each intervention and control arm were pooled using the random effects model and expressed as RR with a 95% confidence interval (CI) [10,11]. If the CI for an estimate includes 1, then it is unable to demonstrate a statistically significant difference between the groups being compared; in contrast, if it does not include 1, then it means that there is a statistically significant difference. For all continuous outcomes (time to hospital discharge (days)), the effect sizes (SMDs) and 95% CIs were determined to perform a comparison between the intervention and control group. The SMD value represents the difference between the means of the two groups divided by a pooled standard deviation. SMDs of approximately 0.2 or less were considered to impose a slight effect, whereas those of approximately 0.5 and 0.8 or greater were considered to impose a moderate and significant effect [10,11,12]. In addition, the heterogeneity of each study was analyzed using the standard coefficient heterogeneity (I^2^) test. Heterogeneity was considered small when I^2^ was less than 25%, moderate when I^2^ was between 25% and 50%, and large when I^2^ was greater than 50%. Subgroup analysis was performed to assess the overall effectiveness based on laboratory parameters and CP transfusion [9,11]. The value *p* < 0.05 was considered statistically significant, and its bias was analyzed using a funnel plot [11].

All these analyses were conducted using Review Manager (version 5.3, Copenhagen, Denmark: The Nordic Cochrane Center, The Cochrane Collaboration, 2014) [13].

### 2.4. Quality Assessment of Included Studies

To explore the validity of eligible RCTs (risk of bias), the quality of biased evaluations was determined by assessing the bias of the random sequence generation, selective reporting, allocation concealment, blinding of participants, blinding of outcome assessment, and incomplete outcome data according to three levels (low, high, and uncertain) following the Cochrane guideline [13] in which low and high risk of bias may indicate a lack of information and uncertainty over the potential for bias, respectively [13].

## 3. Results and Discussion

### 3.1. Characteristics of Included Studies

**Figure 1:**
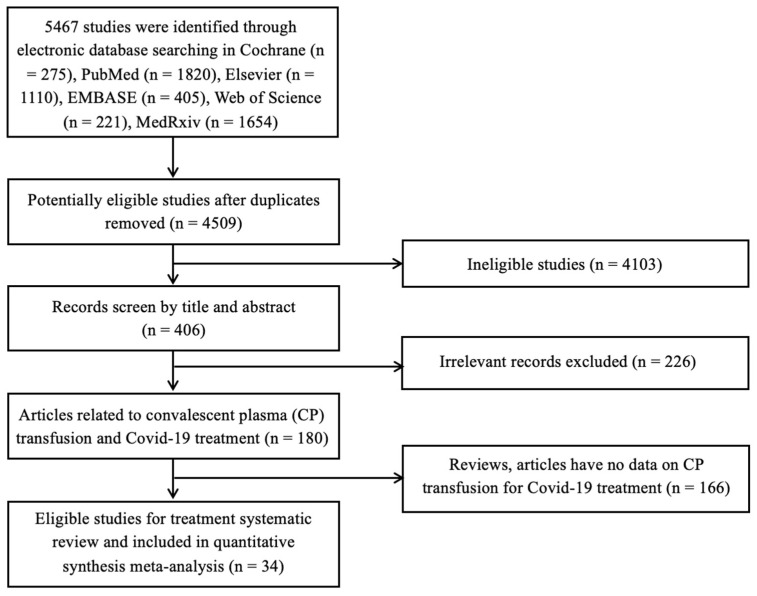
Systematic screening stages of literature review.

Figure 1 shows the flow of qualified articles. Based on searches from the abovementioned databases, 5467 different publications were obtained by screening through their titles and abstracts. After a careful review of these full-text articles pertaining to the clinical efficacy of CP transfusion for the treatment of COVID-19, 34 studies were selected for a quantitative meta-analysis. In particular, this systematic review and meta-analysis focused on evaluating the effectiveness of CP transfusion in clinical improvement, reduction in mortality and length of hospitalization, and infusion-related adverse events. All these 34 included studies investigated the efficacy and safety of CP transfusion for the treatment of COVID-19 infection based on RCTs (Table 1) and provided sufficient data for RR or SMD estimation.

#### Description of Included Studies

Clinical designs, patient characteristics, treatment details, measurement outcomes, and main results of all 34 RCTs randomly assigning COVID-19 patients to CP plus SOC therapy versus SOC or placebo are briefly summarized (Table 1). These trials were conducted in various parts of the world ensuring good geo-ethnic representation, including Europe (n = 12); Asia (n = 7); South America (n = 4); North America (n = 8); and Africa (n = 3). There was a large number of COVID-19 patients (23,550 patients), representing a typical COVID-19 patient population seen in routine clinical practice. CP was administered as a single dose (100–500 mL) [8,14,15,16,17,18,19,20,21,22,23,24,25,26,27,28,29,30,31], two fixed doses (200–300 mL) [8,32,33,34,35,36,37,38,39,40,41], or three fixed doses (200–300 mL) [41,42,43] given 12–36 h apart. Notably, one trial gave a low volume of CP (10 mL) for the first 15 min of administration but allowed transfusion of 100 mL per hour with close monitoring, allowing the infusion rate to be adjusted based on the risk of volume overload and the patient’s tolerance at the discretion of the treating physicians [44]. Additionally, the CP was infused to COVID-19 patients with varying levels of antibody SARS-CoV-2 titers, including very high titer (1:1000–1:3200) [15,21,26,41], high titer (1:160–1:640), and low antibody titer (~1:80) [21,25]. In terms of the control group (SOC treatment or placebo transfusion), while placebo transfusion consisted of 0.9% normal saline buffer, the SOC was in keeping with institutional protocols and national guidelines dictated by the best available evidence at the time and comprised antivirals (oseltamivir, lopinavir/ritonavir, and remdesivir), antimalarials (chloroquine and hydroxychloroquine), immunomodulators (steroids, tocilizumab, and anakinra), broad-spectrum antibiotics, and supportive care (oxygen inhalation and ventilatory support) as appropriate. Almost RCTs among 34 included studies that reported that CP transfusion with high titer of antibody SARS-CoV-2 was neither associated with the improvement in clinical outcomes nor reduction in mortality and length of hospitalization. Even though some adverse events occurred during CP infusion and also SOC or placebo treatment on COVID-19 patients in some RCTs, CP was considered safe to treat COVID-19 disease.

### 3.2. Bias Risk

To validate the quantified randomized studies, the bias risk was evaluated by assessing the biases of the random sequence generation, selective reporting, allocation concealment, blinding of participants, blinding of outcome assessment, and incomplete outcome data corresponding to three levels (low, high, and uncertain) following the Cochrane guidelines [13]. In particular, the low and high risks of bias present insufficient information and uncertainty over the potential for bias, respectively. The results showed that all RCTs were of moderate to good quality with a low risk of bias for the relevant outcomes of interest, except for a high risk of incomplete outcome data (17.7%) due to these RCTs being performed based on different target measurements and the results being presented in various kinds of units (Table 2). It can be seen that the low risks of bias also contributed to improved statistical significance.

### 3.3. Effective and Safety of COVID-19 Treatment Using CP Transfusion

#### 3.3.1. Reducing All-Cause Mortality on Day 28

The association of CP transfusion in reducing all-cause mortality was assessed based on a comparison with the control group (SOC or placebo treatment) in 31 among a total of 34 included RCTs. For instance, Ray et al. (2022) transfused 2 units of 200 mL CP over two consecutive days with a median anti-SARS-CoV-2 IgG titer of 1:250 into 40 patients among 80 participants. They discovered a lower mortality rate on day 28 in the intervention group (20%) compared with the SOC group (35%) [34]. Furthermore, O’Donnell et al. (2021) addressed this aspect in 223 patients with a unit of 300 mL CP transfusion [19]. They observed a significantly reduced in all-cause mortality (12.6% vs. 24.5%) with an RR of 0.51 [0.29; 0.92] between CP and control groups. In contrast, Sekine et al. (2020) revealed that two doses of 300 mL CP plus SOC were not associated with a significantly different risk of mortality as compared with that of SOC groups of 22.5% and 16.3%, respectively [35]. An open-label, randomized trial by Kirenga et al. (2021) of 136 participants also reported that CP did not result in beneficial virological or clinical improvement, especially in the all-cause mortality rate (14.5%), compared with the control arm (11.9%) [14].

**Figure 2:**
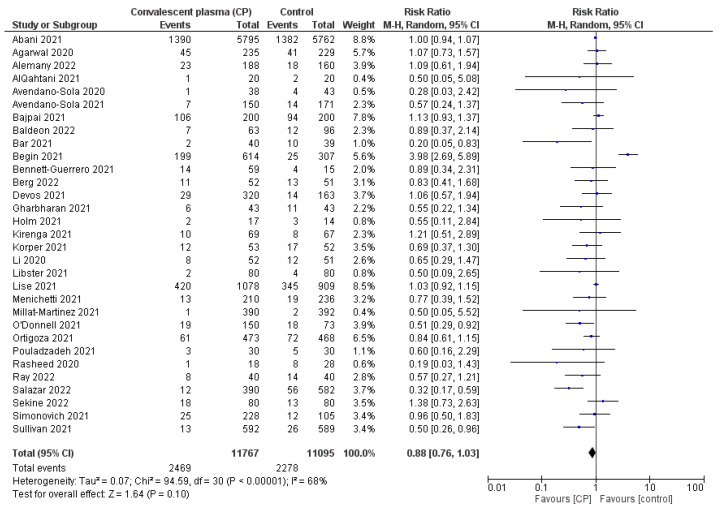
Comparison of reduction in all-cause mortality (number of events) between CP and SOC or placebo groups [8,14,16,17,18,19,20,21,22,24,25,26,27,28,29,31,32,33,34,35,36,37,38,39,40,41,42,43,44,45,46]; (
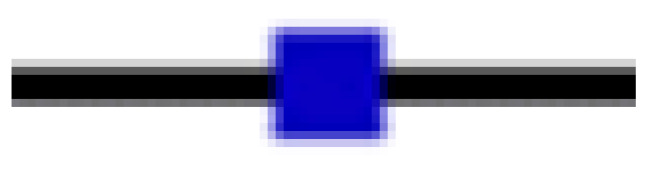
) RR of individual studies; (◆) RR summary of the comparison.

Although almost all RCTs reported positive results in improving the survival of hospitalized patients receiving CP with an RR of 0.88 [0.76; 1.03], the meta-analysis confirmed that there was no significant difference in mortality reduction between CP transfusion and the control group (I^2^ = 68% > 50% and *p* = 0.1 > 0.05) (Figure 2).

#### 3.3.2. Reducing Time to Hospital Discharge

Firstly, the improvement in the clinical symptoms of CP intervention was also indicated by the reduced hospital discharge period of transfused patients. For instance, Korper et al. (2021) measured the improvements in clinical parameters (e.g., total hospitalization days and all cause-mortality) between CP transfusion versus SOC and found that the hospitalization period in the CP group was significantly lower than that of the control group, i.e., 31 and 51 days, respectively [42]. Jordan et al. (2021) also investigated the effectiveness of CP transfusion (300 mL/day) in a total of 86 patients; it showed a negligible difference in the hospital discharge period between the intervention and control groups (−0.96 [−1.41;−0.51]) [22]. Meanwhile, Sekine et al. (2022) reported that CP plus SOC did not result in a shorter hospitalization from the onset of CP administration compared with SOC alone (10 vs. 8 days, respectively) [35]. Pouladzadeh et al. (2021) also revealed that the length of hospital stay did not significantly differ in the CP group compared with controls with 8.7 vs. 6.6 days (*p* > 0.5) [18].

**Figure 3:**
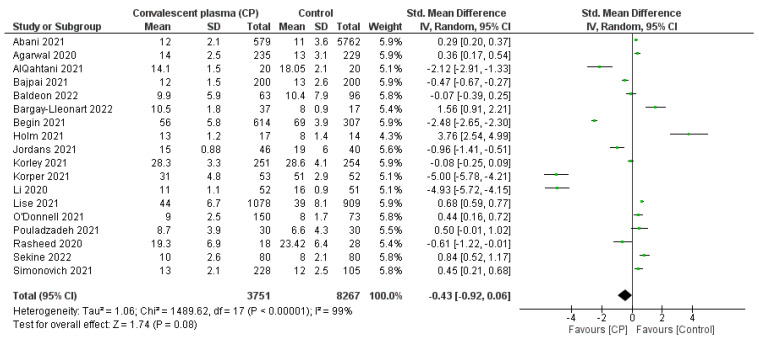
Comparison of reduction in hospitalization length (days) between CP transfusion and control groups [8,14,16,18,19,20,24,25,29,32,36,37,38,41,42,43,45]; (
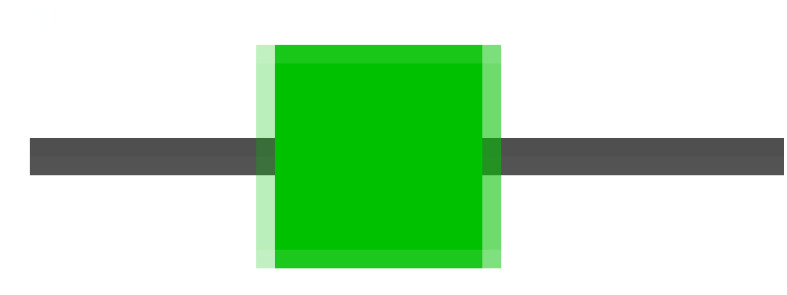
) SMD of individual studies; (◆) SMD summary of the comparison.

To assess the statistical significance of these clinical studies, a comparison of the SMDs of time to hospital discharge (days) was conducted between CP transfusion and the control groups using meta-analysis. Although studies showed a moderate reduction in the hospital discharge period with a total SMD of −0.43 [−0.92; 0.06], the result presented a statistically significant heterogeneity when the SMDs between the two groups were compared (I^2^ = 99% > 50% and *p* = 0.05) (Figure 3). It could be concluded that CP transfusion and the reduced hospitalization length of COVID-19 patients were not correlated.

#### 3.3.3. Adverse Events

**Figure 4:**
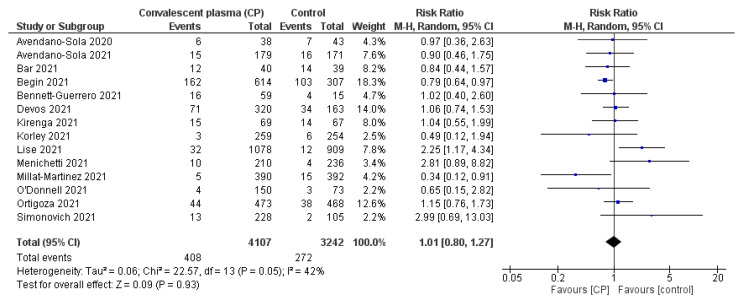
Comparison of adverse event caused by CP transfusion and control groups [15,16,18,20,21,22,27,28,29,34,39,40,41,44]; (
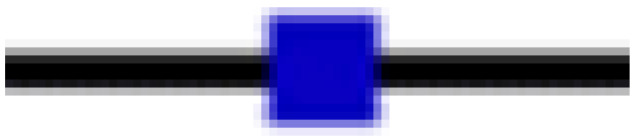
) RR of individual studies; (◆) RR summary of the comparison.

For clinical trial application, the safety of therapy is considered one of the most important points for medical application. In fact, several RCTs have addressed the safety of CP transfusion and reported that side effects occur due to not only CP transfusion but also SOC. For instance, Avendaño-Solà et al. (2021) reported that there were 34 adverse events in 31 patients (15 in the intervention and 16 in the SOC group), of which all adverse events were considered to be related to underlying disease or related complications [26]. Moreover, CP transfusion-related adverse events were reported in cases of worsening dyspnea and transfusion-related acute lung injury [26]. Korley et al. (2021) revealed that adverse events occurred with similar frequency in the CP and placebo groups with the exception of dyspnea, which occurred more frequently in the placebo group, and infusion-related reactions, occurring more frequently in the CP group [15]. A placebo RCT performed by Ortigoza et al. (2021) on 941 patients showed that transfused patients had higher adverse effects (9.4%) than placebo patients (8.2%) [20]. Therefore, a meta-analysis to compare the statistical difference in adverse events (number of events) due to CP transfusion and control groups was performed (Figure 4). The RR of 1.01 [0.80; 1.27] with small heterogeneity I^2^ = 42% (*p* = 0.93) indicated that there was no significant difference in treatment-related toxicity between the two groups. It could be concluded that CP is as safe as SOC and well-tolerated in COVID-19 patients.

#### 3.3.4. Subgroup Analysis

In this study, a subgroup analysis was performed to evaluate the efficacy of CP transfusion in reducing the mortality of COVID-19 patients corresponding to six sub-criteria (Table S1). First, the subgroup analysis was stratified by countries, pooling high-income countries (Australia, Bahrain, Belgium, Chile, Germany, Italy, Netherlands, Spain, United States, United Kingdom, and South Africa) versus middle-income countries (Argentina, China, India, Iran, Iraq, Ecuador, and Brazil). Secondly, the included RCTs were classified based on the level of antibody SARS-CoV-2 titer (high and low) and types of antibodies (SARS-CoV-2 neutralizing antibodies and SARS-CoV-2 IgG antibodies) that were confirmed by each RCT. Thirdly, a sample size of RCTs was also considered for the subgroup analysis, and an RCT was defined as a large size if it included more than 200 participants versus a small size with below 200 participants. Fourthly, RCTs were categorized based on the design of control groups, including placebo transfusion and SOC. Finally, it was analyzed according to the severity of COVID-19 disease in patients at the onset of RCTs.

**Figure 5:**
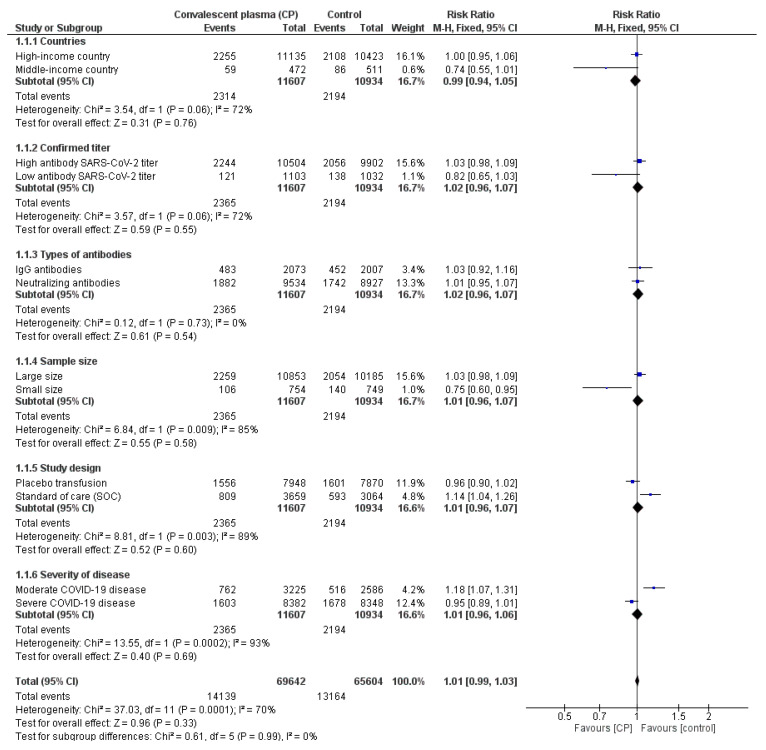
Subgroup analysis for mortality reduction based on six sub-criteria: country, confirmed titer, types of antibodies, sample size, study design, and severity of disease.

Even though the difference between CP transfusion and SOC or placebo treatment in reducing mortality was compared based on subgroup analysis, it was confirmed that there was no significant difference between these two groups with an overall RR of 1.01 [0.99; 1.03] (*p* = 0.33 and I^2^ = 70% > 50%) (Figure 5). When considering the small group analysis, CP infusion could effectively reduce mortality in RCTs using either SOC treatment for the control arm or treatment for mild-to-moderate COVID-19 patients with an RR of 1.14 [1.04; 1.26] and 1.18 [1.07; 1.31], respectively.

### 3.4. Analysis Bias

**Figure 6:**
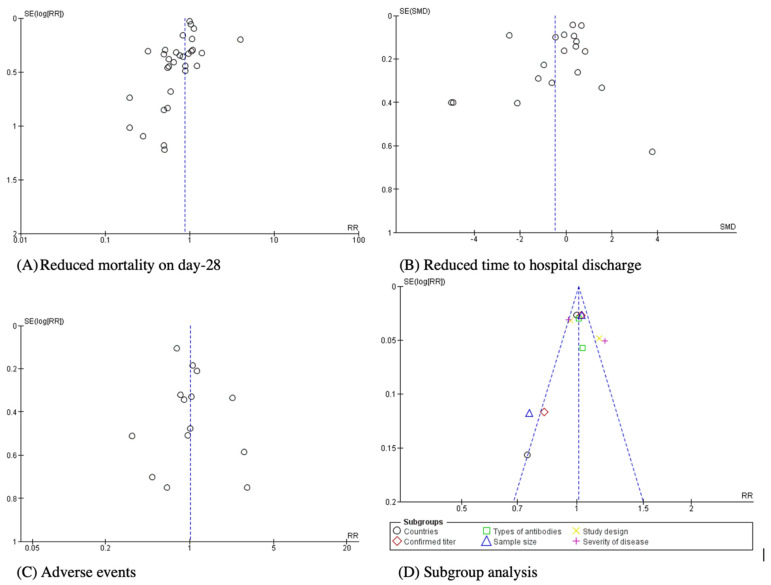
Funnel plots of included RCTs for investigating efficacy of CP transfusion against COVID-19, compared with SOC or placebo treatment.

Funnel plots were constructed to detect the publication bias (Figure 6). The results indicated possible publications almost existed in the outcome. All the funnel plots were symmetrical and tended to include not only positive outcomes but also a number of negative outcomes of each aspect analyzed. Therefore, it is suggested that this systematic review and meta-analysis summarized sufficient and high-quality RCTs, thereby reducing publication bias and increasing the statistical significance of the whole meta-analysis.

## 4. Discussion

In the early days of the COVID-19 pandemic, CP transfusion was considered one of the effective therapies to improve clinical symptoms and reduce mortality caused by COVID-19. Despite the lack of definitive evidence of efficacy and safety, CP was granted by the United States FDA in large in August 2020. According to safety data, 20,000 patients initially and then over 35,000 hospitalized patients in the United States reported a very low incidence (<1%) of adverse events related to transfusion (acute lung injury, allergic reactions, and circulatory overload), which in the first few hours was no different from transfusions [47]. In order to provide accurate evidence for the efficacy and safety of CP transfusion, this updated systematic review and meta-analysis was designed to summarize and analyze the outcomes extracted from RCTs on CP therapy. According to eligibility criteria, 34 RCTs with a total of 23,550 participants were enrolled and evaluated, and the results showed that the addition of CP to the current SOC was not associated with a statistically significant reduction in mortality and length of hospitalization with a low risk of bias. Furthermore, the risk of side effects related to CP transfusion was negligible and did not differ significantly from SOC or placebo treatment, suggesting that CP was safe and well-tolerated for COVID-19 patients.

Even though there was no statistical significance in the efficacy of CP transfusion for COVID-19 patients between these RCTs, it could establish some useful recommendations for improving the clinical outcomes of using CP therapy through individual RCTs. Firstly, there is some suggestion of a dose–response relationship, as those who receive plasma units with high titers of neutralizing antibodies have a lower mortality rate than patients receiving units with lower titers [14,25,29]. Secondly, CP may benefit select populations, especially those with comorbidities who are treated with early CP (within few days of symptom onset and/or disease of mild to moderate severity) and also help to reduce the progression of severity of COVID-19 compared to delayed or deferred transfusion (>7 days of symptom onset and/or severe to critical illness) [14,16,21,38]. For example, Baldeon et al., 2022 confirmed that the early use of CP could decrease the length of hospital stay and improve respiratory function [23]. Rasheed et al. (2020) revealed that CP was an effective therapy if there were donors with high level of SARS-CoV-2 antibodies and if patients were at their early stage of critical illness, being no more than 3 days from onset of symptoms [46].

Recently, there have been a number of meta-analyses dealing with this topic designed to analyze data extracted from several kinds of studies, including RCTs, cohort-match control, individual studies, and also unpublished studies. For example, Gupta et al., 2021 also designed a meta-analysis based on only RCTs but with a small number of eligible studies (12 RCTs; 13,260 patients) and evaluated the results based on RR value according to the Cochrane methodology [48]. This study reported that there was no significant difference in clinical improvement rate (RR = 1.00 [0.98; 1.02], *p* = 0.96) and also in the reduction in mortality (RR = 0.81 [0.65; 1.02], *p* = 0.08); however, the incidence of serious infusion-related adverse events was low (3.25%). It concluded that the addition of CP to SOC therapy was generally safe but did not provide any significant clinical benefit or mortality reduction in COVID-19 [48]. On the other hand, Axfors et al. (2021) conducted a meta-analysis of 33 trials involving a total of 16,477 patients, including ongoing, discontinued, and completed RCTs that compared CP with placebo or no treatment in COVID-19 patients, regardless of setting or treatment schedule. According to the obtained RR of 0.97 [0.92; 1.02] and heterogeneity (I^2^ = 0%), it was suggested that CP transfusion should not be used outside of randomized trials because this therapy did not improve the survival of COVID-19 patients [47]. In contrast, the study of Kloypan et al. (2021) included 18 peer-reviewed clinical trials, 3 preprints, and 26 observational studies and then used the random-effect model to estimate the clinical benefits of CP infusion [49]. The comparisons between CP and SOC or placebo group of 18 peer-reviewed trials and also 26 observational studies found that CP was associated with a reduced risk of all-cause mortality in critically ill COVID-19 patients [49]. It can be seen that all the current meta-analyses were performed with small numbers of RCTs that were not large enough to accurately estimate the size effect as well as the significant difference between CP versus SOC or placebo treatment, leading to the underestimation of several points of the meta-analysis, such as all-cause mortality and adverse events. In this study, we described only meta-analysis with a collaborative approach to collecting published randomized trial evidence regardless of study area and design. This study consisted of a largest dataset (23,550 patients) derived from only RCTs and also provides comprehensive information on clinical trials such as doses of administration, the severity of disease, SARS-CoV-2 antibody titers, region of study, and specific measurement outcomes, expecting to provide highly accurate analytical results and improve the quality of evidence for the application of CP in clinical trials compared to the traditional systematic review and meta-analysis. Thereby, this study is expected to complement traditional systematic reviews and meta-analyses.

Moreover, although RCTs in thousands of non-severe, severe, and critical COVID-19 patients have been done to provide evidence of benefit for CP in the treatment of COVID-19, WHO has reported that CP does not improve survival or reduce the need for mechanical ventilation, while it has significant costs. Therefore, the use of CP to treat COVID-19 should not be continued beyond clinical trials because this therapy does not bring significant benefits for improving the survival rates of COVID-19 patients.

## 5. Limitations of Study

The meta-analysis performed presents four main limitations. Firstly, although a large number of RCTs were included, data for some of the analyses were lacking, so some comparisons, particularly regarding adverse events, could only be performed with small amounts of data, resulting in underestimation results. Secondly, most of the included studies were conducted under different treatment conditions (e.g., the dosages and time point of CP transfusion, the period from symptom onset to CP administration, and severity of disease) and patient characteristics; therefore, the meta-analysis evaluations may not be highly accurate. Thirdly, we were unable to access the potential effects of time, dose, or titer for CP infusion. We also did not collect details about various patient characteristics including age, sex, and treatments that were simultaneously disclosed in individual trial publications to allow for a better insight on larger potential benefits in certain subgroups. Fourthly, our subgroup analysis in some cases used arbitrary classifications, although chosen to be consistent with clinical practice, such as for plasma antibody titers and study design. We considered all subgroup analyses to be exploratory, and caution should be exercised in interpreting those results.

## 6. Conclusions

The recent COVID-19 pandemic remains an emerging issue that has not yet been resolved using specific drugs or medical therapies. Among the conventional treatments, CP transfusion has been considered an adjuvant therapy approved by the FDA to treat patients with severe COVID-19 symptoms. According to consolidated clinical data derived from 34 studies that included 23,550 patients with the disease, the CP therapy provided no benefit for COVID-19 compared with SOC or placebo treatment in all relevant points, including reduced mortality and length of hospitalization. In addition, although adverse reactions during the intervention therapy have been reported in some cases of RCTs, CP transfusion was considered to be as safe as SOC treatment which has been approved by medical institutions. Since the number of RCTs can increase dramatically in a short period of time, updated systematic reviews and meta-analyses need to be repeated to evaluate a larger dataset and then obtain an up-to-date statistical result on this topic.

## Figures and Tables

**Table 1:** Summary of the characteristics of included studies.

Author	Region	No. of Patients	Age (Years)	Clinical Design	Treatment Arm	CP Transfusion	Measurement Outcomes	Main Results
Abani et al. 2021 [36]	United Kingdom	11558	~63	Open-label RCT	CP transfusion Placebo transfusion	Two units of 275 mL CP on consecutive 2 days Median SARS-CoV-2 neutralizing antibodies titer of 1:400	All-cause mortality on day 28 Time to hospital discharge	High-titer CP did not improve survival or other prespecified clinical outcomes
Agarwal et al. 2020 [8]	India	464	~52	Open label, parallel arm, phase II, multicenter RCT	CP transfusion plus SOC SOC	Two units of 200 mL CP, transfused 24 h apart SARS-CoV-2 neutralizing antibodies titer > 1:160	All-cause mortality on day 28 Time to hospital discharge	CP was not associated with a reduction in progression to severe COVID-19 or all-cause mortality
Alemany et al. 2022 [45]	Spain	376	~56	Multicenter, double-blind, placebo-controlled RCT	CP transfusion Placebo transfusion	One unit of 250 mL CP Median SARS-CoV-2 neutralizing antibodies titer > 1:250	All-cause mortality on day 28	CP did not prevent progression from mild to severe illness and did not reduce viral load in outpatients with COVID -19
AlQahtani et al. 2021 [37]	Bahrain	40	~52	Prospective, open-label RCT	CP transfusion plus SOC SOC	Two units of 200 mL CP over 2 h over 2 successive days Median SARS-CoV-2 neutralizing antibodies titer of 84.95 AU/mL	All-cause mortality on day 28 Time to hospital discharge	There were no significant differences in the primary and secondary outcome measures between the two groups
Avendano-sola et al. 2020 [21]	Spain	81	~59	Multicenter RCT	CP transfusion plus SOC SOC	One unit of 250–300 mL CP SARS-CoV-2 neutralizing antibodies titer > 1:80	All-cause mortality on day 28 Adverse events	No significant differences were found in secondary endpoints
Avendano-sola et al. 2021 [26]	Spain	350	~62	Open-label RCT	CP transfusion plus SOC SOC	One unit of 250–300 mL CP Median SARS-CoV-2 neutralizing antibodies of 1:157	All-cause mortality on day 28 Adverse events	CP showed a significant benefit in preventing progression to noninvasive ventilation or high-flow oxygen or death at 28 days.
Bajpai et al. 2022 [31]	India	400	~55	Open-label, multicenter, phase-III RCT	CP transfusion plus SOC SOC	Two units of 250 mL CP on two consecutive days Median SARS-CoV-2 neutralizing antibodies titer ≥ 1:640	All-cause mortality on day 28 Time to hospital discharge	CP should be transfused in COVID-19 patients along with SOC in the initial 3 days of hospitalization for better clinical outcomes
Baldeon et al. 2022 [23]	Ecuador	158	~56	Double-blind, placebo-controlled RCT	CP transfusion plus SOC SOC	One unit of 100 mL CP Median SARS-CoV-2 neutralizing antibodies titer > 1:1350	All-cause mortality on day 28 Time to hospital discharge	CP was safe and its early use could decrease the length of hospital staying and improve respiratory function
Bar et al. 2021 [38]	United States	79	~63	Open-label RCT	CP transfusion plus SOC SOC	Two units of 200 mL CP on the 1st day of administration Median SARS-CoV-2 neutralizing antibodies of 8.189 AU/mL	All-cause mortality on day 28 Adverse events	CP was generally safe and well-tolerated
Bargay-Lleonart et al. 2022 [24]	Balearic Islands	54	~59	Open-label, multicenter RCT	CP transfusion Placebo transfusion	Two units of 300 mL CP within 48 h Median SARS-CoV-2 IgG antibodies titer > 1:160	Time to hospital discharge	CP was a safe therapy for COVID-19 treatment, and could effectively help restore physical condition earlier than the standard treatment
Begin et al. 2021 [28]	Canada	921	~69	Open-label RCT	CP transfusion plus SOC SOC	One unit of 500 mL CP Median SARS-CoV-2 neutralizing antibodies titer > 1:160	All-cause mortality on day 28 Time to hospital discharge Adverse events	CP did not reduce the risk of death at 30 days in hospitalized patients
Bennett-Guerrero et al. 2021 [39]	United States	74	~67	Double-blind RCT	CP transfusion plus SOC SOC	Two units of total volume 480 mL CP Median SARS-CoV-2 neutralizing antibodies titer of 1:526	All-cause mortality on day 28 Adverse events	CP administration increased antibodies to severe acute respiratory syndrome COVID-19 disease but was not associated with improved outcome
Berg et al. 2022 [30]	South Africa	103	~56	Double-blinded, multicenter RCT	CP transfusion Placebo transfusion	One unit of 200 mL CP Median SARS-CoV-2 neutralizing antibodies titer > 1:160	All-cause mortality on day 28	CP transfusion effectively reduced progression to severe COVID-19 among older people
Devos et al. 2022 [33]	Belgium	483	~62	Open-label, multicenter RCT	CP transfusion plus SOC SOC	Two units of 200–250 mL CP with 36 h Median SARS-CoV-2 neutralizing antibodies titer > 1:320	All-cause mortality on day 28 Adverse effects	Transfusion of CP with high titer early did not result in a significant improvement in clinical status or reduced mortality
Gharbharan et al. 2021 [25]	Netherlands	86	~62	RCT	CP transfusion plus SOC SOC	Two units of 300 mL CP within 5 days Median SARS-CoV-2 neutralizing antibodies titer of 1:160	All-cause mortality on day 28	CP did not improve clinical outcome 10 days after symptom onset
Holm et al. 2021 [41]	Netherlands	31	~60	Open-label RCT	CP transfusion plus SOC SOC	Three units of 200–250 mL CP during 30 min on 3 consecutive days Median SARS-CoV-2 neutralizing antibodies titer of 1:116	All-cause mortality on day 28 Time to hospital discharge	CP did not improve clinical outcome after the treatment period.
Jordans et al. 2021 [22]	Netherlands	86	~63	Multicenter open-label RCT	CP transfusion plus SOC SOC	One unit of 300 mL CP Median SARS-CoV-2 neutralizing antibodies titer > 1:160	Time to hospital discharge	CP treatment did not improve survival or disease course, nor did it alter relevant virological and immunological parameters
Kirenga et al. 2021 [14]	Uganda	136	~50	Open-label RCT	CP transfusion plus SOC SOC	One unit of 300 mL CP. Median IgG anti-SARS-CoV-2 titer of 1:160	All-cause mortality on day 28 Adverse events	CP did not result in beneficial virological or clinical improvements
Korley et al. 2021 [15]	United States	511	~54	Multicenter, single-blind RCT	CP transfusion Placebo transfusion	One unit 250 mL of CP Median SARS-CoV-2 neutralizing antibodies 2 titer of 1:641	Time to hospital discharge Adverse events	CP transfusion did not prevent disease progression Adverse effects often occurred with dyspnea in the placebo group compared to CP group
Korper et al. 2021 [42]	Germany	105	~62	Open-label, multicenter RCT	CP transfusion plus SOC SOC	Three units of total volume 846 mL CP Median SARS-CoV-2 neutralizing antibodies titer of 1:160	Clinical improvement All-cause mortality on day 28 Time to hospital discharge	CP added to standard treatment was not associated with a significant improvement in the primary and secondary outcomes
Li et al. 2020 [44]	China	103	~70	Open-label, multicenter RCT	CP transfusion plus SOC SOC	One unit of 10 mL for the 1st 15 min, then 100 mL per hour with close monitoring SARS-CoV-2 IgG antibodies titer ≥ 1:640	All-cause mortality on day 28 Time to hospital discharge	CP treatment did not result in a statistically significant improvement in time to clinical improvement within 28 days Interpretation was limited by early termination of the trial
Libster et al. 2021 [29]	Argentina	160	~75	Double-blind, placebo-controlled RCT	CP transfusion Placebo transfusion	One unit of 250 mL CP over a period of 1.5–2 h SARS-CoV-2 IgG antibodies titer > 1:1000	All-cause mortality on day 28	Early administration of CP reduced the progression of COVID-19 No solicited adverse events were observed
Lise et al. 2021 [40]	United Kingdom	1988	~61	Multicenter, open-label RCT	CP transfusion plus SOC SOC	Two units of total volume 250 mL CP Median SARS-CoV-2 IgG antibodies titer of 1:160	All-cause mortality on day 28 Time to hospital discharge Adverse events	Only 1 event was considered to be possibly or probably related to convalescent plasma CP had a low likelihood of providing improvement in the number of organ support-free days
Menichetti et al. 2021 [43]	Italia	447	~64	Open-label RCT	CP transfusion plus SOC SOC	Three units of 200 mL CP over a period of 2 h daily SARS-CoV-2 neutralizing antibodies titer ≥1:160	All-cause mortality on day 28 Adverse events	CP did not reduce the progression to respiratory failure or death within 30 days among these patients vs. those receiving standard treatment
Millat-Martinez et al. 2021 [27]	Spain	782	~58	Two-double blind RCT	CP transfusion plus SOC SOC	One unit of 300 mL CP Median SARS-CoV-2 neutralizing antibodies titer > 1:320	All-cause mortality on day 28 Adverse events	Treatment with CP did not improve the outcome of COVID-19 patients
O’Donnell et al. 2021 [19]	United States	223	~70	Double-blind RCT	CP transfusion Placebo transfusion	One unit of 300 mL CP Median SARS-CoV-2 neutralizing antibodies titer of 1:160	All-cause mortality on day 28 Time to hospital discharge Adverse events	No significant improvement in the clinical scale at day 28 CP was associated with significantly improved survival
Ortigoza et al. 2021 [20]	United States	941	~63	Double-blind, placebo-controlled RCT	CP transfusion Placebo transfusion	One unit of 250 mL CP within 24 h Median SARS-CoV-2 neutralizing antibodies titer of 1:175	All-cause mortality on day 28 Adverse events	CP did not meet the prespecified outcomes for CP efficacy. The high-titer CP may have benefited participants early in pandemic
Pouladzadeh et al. 2021 [18]	Iran	60	~63	Parallel-group, single-blind, and RCT	CP transfusion plus SOC SOC	One unit of 500 mL CP on the admission day Median SARS-CoV-2 IgG antibodies titer > 1:160	All-cause mortality on day 28 Time to hospital discharge	CP therapy did not have any serious side effects on patients CP did not considerably affect the mortality rate
Rasheed et al. 2020 [46]	Iraq	49	~60	Open-label RCT	CP transfusion plus SOC SOC	One unit of 400 mL CP over 2 h SARS-CoV-2 IgG antibodies titer ≥ 1:160	All-cause mortality on day 28 Time to hospital discharge	CP therapy was an effective therapy if there were donors with high level of SARS-CoV-2 antibodies, and if recipients were at their early stage of critical illness
Ray et al. 2022 [34]	India	80	~60	Open label, single center, phase II RCT	CP transfusion plus SOC SOC	Two units of 200 mL CP on 2 consecutive days Median SARS-CoV-2 neutralizing antibodies titer of 1:250	All-cause mortality on day 28	No adverse effect was reported with CP treatment. The CP treatment did not improve the survival of patients
Salazar et al. 2021 [32]	United States	903	~65	Open-label RCT	CP transfusion Placebo transfusion	Two units of 300 mL CP within 74 h Median SARS-CoV-2 neutralizing antibodies titer > 1:1350	All-cause mortality on day 28	CP transfusion of COVID-19 patients soon after hospitalization with high-titer anti-spike protein RBD IgG present in convalescent plasma significantly reduced mortality
Sekine et al. 2022 [35]	Brazil	160	~60.5	Open-label, parallel RCT	CP transfusion plus SOC SOC	Two units of 300 mL CP within 48 h Median SARS-CoV-2 neutralizing antibodies titer of 1:320	All-cause mortality on day 28 Time to hospital discharge	CP with SOC did not result in a higher proportion of clinical improvement on day 28 in hospitalized patients
Simonovich et al. 2021 [17]	Argentina	333	~62	Open-label, RCT	CP transfusion Placebo transfusion	One unit of a median 500 mL CP Median SARS-CoV-2 neutralizing antibodies titer of 1:3200	All-cause mortality on day 28 Time to hospital discharge Adverse events	At day 30, no significant difference was noted between 2 groups Adverse events and serious adverse events were similar in the two group
Sullivan et al. 2021 [16]	United States	1225	~44	Multicenter, double-blind RCT	CP transfusion Placebo transfusion	One unit of 250 mL CP Median SARS-CoV-2 IgG antibodies titer > 1:160	All-cause mortality on day 28	High-titer CP was an effective early outpatient COVID-19 treatment with the advantages of low cost, wide availability, and rapid resilience

**Table 2:** Bias risk rating for each RCT.

References	Random Sequence Generation	Allocation Concealment	Selective Reporting	Blinding of Participants	Blinding of Outcome Assessment	Incomplete Outcome Data
Abani et al. 2021 [36]						
Agarwal et al. 2020 [8]						
Alemany et al. 2022 [45]						
AlQahtani et al. 2021 [37]						
Avendano-sola et al. 2020 [21]						
Avendano-sola et al. 2021 [26]						
Bajpai et al. 2022 [31]						
Baldeon et al. 2022 [23]						
Bar et al. 2021 [38]						
Bargay-Lleonart et al. 2022 [24]						
Begin et al. 2021 [28]						
Bennett-Guerrero et al. 2021 [39]						
Berg et al. 2022 [30]						
Devos et al. 2022 [33]						
Gharbharan et al. 2021 [25]						
Holm et al. 2021 [41]						
Jordans et al. 2021 [22]						
Kirenga et al. 2021 [14]						
Korley et al. 2021 [15]						
Korper et al. 2021 [42]						
Li et al. 2020 [44]						
Libster et al. 2021 [29]						
Lise et al. 2021 [40]						
Menichetti et al. 2021 [43]						
Millat-Martinez et al. 2021 [27]						
O’Donnell et al. 2021 [19]						
Ortigoza et al. 2021 [20]						
Pouladzadeh et al. 2021 [18]						
Rasheed et al. 2020 [46]						
Ray et al. 2022 [34]						
Salazar et al. 2021 [32]						
Sekine et al. 2022 [35]						
Simonovich et al. 2021 [17]						
Sullivan et al. 2021 [16]						
Total low risk of bias (%)	73.5	79.4	82.4	82.4	79.4	79.4
Total high risk of bias (%)	2.9	11.8	5.9	5.9	8.8	17.7
Total uncertain risk of bias (%)	23.6	8.8	11.7	11.7	11.8	2.9

## Supplementary Materials

The following supporting information can be downloaded at: https://www.mdpi.com/article/10.3390/ijerph191710622/s1, Table S1: Characteristics of RCTs for subgroup analysis.
