# Supplementary material for: Efficacy and Safety of COVID-19 Treatment Using Convalescent Plasma Transfusion: Updated Systematic Review and Meta-Analysis of Randomized Controlled Trials
Source: Int J Environ Res Public Health. 2022 Aug 25;19(17):10622. doi: 10.3390/ijerph191710622 (PMC9518594; doi:10.3390/ijerph191710622)
Supplement: Supplementary file 1 [file ijerph-19-10622-s001.zip › ijerph-1849323-supplementary.pdf]

Supporting information

Efficacy and safety of COVID-19 treatment using convalescent plasma transfusion: Updated systematic review and meta-analysis of randomized controlled trials

Hyun-Jun Lee[1,†], Jun-Hyeong Lee[1,†], Yejin Cho[1,†], Le Thi Nhu Ngoc[2,*,] Young-Chul Lee [1,*]

[1]Department of BioNano Technology, Gachon University, 1342 Seongnam-Daero, Sujeong-Gu, Seongnam-Si, Gyeonggi-Do 13120, Republic of Korea

[2]Department of Industrial and Environmental Engineering, Graduate School of Environment, Gachon University, 1342 Seongnam-Daero, Sujeong-Gu, Seongnam-Si, Gyeonggi-Do 13120, Republic of Korea

[*]Correspondence: dreamdbs@gachon.ac.kr (Y.-C.L), nhungocle92@gmail.com (L.T.N.N), Tel: +82-31-750-8751 (Y-C.L), Fax: +82-31-755-7405 (Y-C.L.)

† These authors contributed equally to this work

Table S1. Characteristics of RCTs for subgroup analysis.

Study	Regions	Severity of disease	Confirmed titer	Types of antibodies	Sample size	Study design	No. of events		No. of total participants	
							CP	Control	CP	Control
Abani *et al.* 2021 [37]	High-income country	Severe COVID-19 disease	High titer	SARS-CoV-2 neutralizing antibodies	Large	Placebo	1390	5795	1382	5762
Agarwal *et al.* 2020 [8]	Middle-income country	Moderate COVID-19 disease	Low titer	SARS-CoV-2 neutralizing antibodies	Large	SOC	45	235	41	229
Alemany *et al.* 2022 [46]	High-income country	Moderate COVID-19 disease	High titer	SARS-CoV-2 neutralizing antibodies	Large	Placebo	23	188	18	160
AlQahtani *et al.* 2021 [38]	High-income country	Severe COVID-19 disease	High titer	SARS-CoV-2 neutralizing antibodies	Small	SOC	1	20	2	20
Avendano-sola *et al.* 2020 [22]	High-income country	Severe COVID-19 disease	Low titer	SARS-CoV-2 neutralizing antibodies	Small	SOC	1	38	4	43
Avendano-sola *et al.* 2021 [27]	High-income country	Severe COVID-19 disease	High titer	SARS-CoV-2 neutralizing antibodies	Large	SOC	7	150	14	171
Bajpai *et al.* 2022 [32]	Middle-income country	Severe COVID-19 disease	High titer	SARS-CoV-2 neutralizing antibodies	Large	SOC	106	200	94	200
Baldeon *et al.* 2022 [24]	Middle-income country	Moderate COVID-19 disease	High titer	SARS-CoV-2 neutralizing antibodies	Small	SOC	7	63	12	96
Bar *et al.* 2021 [39]	High-income country	Severe COVID-19 disease	High titer	SARS-CoV-2 neutralizing antibodies	Small	SOC	2	40	10	39

Begin *et al.* 2021 [29]	High-income country	Moderate COVID-19 disease	High titer	SARS-CoV-2 neutralizing antibodies	Large	SOC	199	614	25	307
Bennett-Guerrero *et al.* 2021 [40]	High-income country	Severe COVID-19 disease	High titer	SARS-CoV-2 neutralizing antibodies	Small	SOC	14	59	4	15
Berg *et al.* 2022 [31]	High-income country	Moderate COVID-19 disease	Low titer	SARS-CoV-2 neutralizing antibodies	Small	Placebo	11	52	13	51
Devos *et al.* 2022 [34]	High-income country	Moderate COVID-19 disease	High titer	SARS-CoV-2 neutralizing antibodies	Large	SOC	29	320	14	163
Gharbharan *et al.* 2021 [26]	High-income country	Severe COVID-19 disease	High titer	SARS-CoV-2 neutralizing antibodies	Small	SOC	6	43	11	43
Holm *et al.* 2021 [42]	High-income country	Severe COVID-19 disease	Low titer	SARS-CoV-2 neutralizing antibodies	Small	SOC	2	17	3	14
Kirenga *et al.* 2021 [15]	Middle-income country	Severe COVID-19 disease	High titer	SARS-CoV-2 IgG antibodies	Small	SOC	10	69	8	67
Korper *et al.* 2021 [43]	High-income country	Moderate COVID-19 disease	Low titer	SARS-CoV-2 neutralizing antibodies	Small	SOC	12	53	17	52
Li *et al.* 2020 [45]	Middle-income country	Severe COVID-19 disease	High titer	SARS-CoV-2 IgG antibodies	Small	SOC	8	52	12	51
Libster *et al.* 2021 [30]	Middle-income country	Severe COVID-19 disease	High titer	SARS-CoV-2 IgG antibodies	Small	Placebo	2	80	4	80
Lise *et al.* 2021 [41]	High-income country	Moderate COVID-19 disease	High titer	SARS-CoV-2 IgG antibodies	Large	SOC	420	1078	345	909
Menichetti *et al.* 2021 [44]	High-income country	Severe COVID-19 disease	High titer	SARS-CoV-2 neutralizing antibodies	Large	SOC	13	210	19	236
Millat-Martinez *et al.* 2021 [28]	High-income country	Severe COVID-19 disease	Low titer	SARS-CoV-2 neutralizing antibodies	Large	SOC	1	390	2	392

O'Donnell *et al.* 2021 [20]	High-income country	Severe COVID-19 disease	Low titer	SARS-CoV-2 neutralizing antibodies	Large	Placebo	19	150	18	73
Ortigoza *et al.* 2021 [21]	High-income country	Severe COVID-19 disease	High titer	SARS-CoV-2 neutralizing antibodies	Large	Placebo	61	473	72	468
Pouladzadeh *et al.* 2021 [19]	Middle-income country	Moderate COVID-19 disease	Low titer	SARS-CoV-2 IgG antibodies	Small	SOC	3	30	5	30
Rasheed *et al.* 2020 [14]	Middle-income country	Severe COVID-19 disease	Low titer	SARS-CoV-2 IgG antibodies	Small	SOC	1	18	8	28
Ray *et al.* 2022 [35]	Middle-income country	Severe COVID-19 disease	Low titer	SARS-CoV-2 neutralizing antibodies	Small	SOC	8	40	14	40
Salazar *et al.* 2021 [33]	High-income country	Severe COVID-19 disease	High titer	SARS-CoV-2 neutralizing antibodies	Large	Placebo	12	390	56	582
Sekine *et al.* 2022 [36]	Middle-income country	Severe COVID-19 disease	Low titer	SARS-CoV-2 neutralizing antibodies	Small	SOC	18	80	13	80
Simonovich *et al.* 2021 [18]	Middle-income country	Severe COVID-19 disease	High titer	SARS-CoV-2 neutralizing antibodies	Large	Placebo	25	228	12	105
Sullivan *et al.* 2021 [17]	High-income country	Moderate COVID-19 disease	High titer	SARS-CoV-2 IgG antibodies	Large	Placebo	13	592	26	589
